# Is the positive association between middle-income and rich household wealth and adult sub-Saharan African women’s overweight status modified by the level of education attainment? A cross-sectional study of 22 countries

**DOI:** 10.1186/s12889-020-08956-3

**Published:** 2020-06-25

**Authors:** Ifeoma D. Ozodiegwu, Henry V. Doctor, Megan Quinn, Laina D. Mercer, Ogbebor Enaholo Omoike, Hadii M. Mamudu

**Affiliations:** 1 Institute for Global Health, Feinberg School of Medicine, Northwestern University, Chicago, IL USA; 2 Department of Science, Information, and Dissemination, World Health Organization, Regional Office for the Eastern Mediterranean, Cairo, Egypt; 3 Institute for Disease Modeling, Bellevue, Washington USA; 4 Current Address: PATH, Seattle, USA; 5 Department of Health Services Management and Policy, East Tennessee State University, Johnson City, Tennessee USA

**Keywords:** Overweight, Sub-Saharan Africa, Socioeconomic status, Effect modification, Educational attainment, Household wealth

## Abstract

**Background:**

Previous studies show a positive association between household wealth and overweight in sub-Saharan African (SSA) countries; however, the manner in which this relationship differs in the presence of educational attainment has not been well-established. This study examined the multiplicative effect modification of educational attainment on the association between middle-income and rich household wealth and overweight status among adult females in 22 SSA countries. We hypothesized that household wealth was associated with a greater likelihood of being overweight among middle income and rich women with lower levels of educational attainment compared to those with higher levels of educational attainment.

**Methods:**

Demographic and Health Survey (DHS) data from 2006 to 2016 for women aged 18–49 years in SSA countries were used for the study. Overweight was defined as a body mass index (BMI) ≥ 25 kg/m^2^. Household wealth index tertile was the exposure and educational attainment, the effect modifier. Potential confounders included age, ethnicity, place of residence, and parity. Descriptive analysis was conducted, and separate logistic regression models were fitted for each of the 22 SSA countries to compute measures of effect modification and 95% confidence intervals. Analysis of credibility (AnCred) methods were applied to assess the intrinsic credibility of the study findings and guide statistical inference.

**Results:**

The prevalence of overweight ranged from 12.6% in Chad to 56.6% in Swaziland. Eighteen of the 22 SSA countries had measures of effect modification below one in at least one wealth tertile. This included eight of the 12 low-income countries and all 10 middle income countries. This implied that the odds of overweight were greater among middle-income and rich women with lower levels of educational attainment than those with higher educational attainment. On the basis of the AnCred analysis, it was found that the majority of the study findings across the region provided some support for the study hypothesis.

**Conclusions:**

Women in higher wealth strata and with lower levels of educational attainment appear to be more vulnerable to overweight compared to those in the same wealth strata but with higher levels of educational attainment in most low- and middle- income SSA countries.

## Background

Evidence from multiple country studies indicate that women of higher socioeconomic status (SES), as defined by household wealth are more likely to be overweight or obese in sub-Saharan African countries. [1]. High affluent groups in African countries have been found to have lower levels of physical activity and to consume more processed food, which may explain their elevated adiposity risk [2]. Sociocultural factors such as the association of larger bodies with higher social status in African culture, coupled with other global factors - unregulated marketing of obesogenic foods and greater levels of mechanization – and the abundance of resources conferred by high income status may result in increased consumption of unhealthy foods and sedentarism [3, 4]. Educational attainment have been found to modify the income-health relationship, that is, individuals with more years of schooling have better health outcomes at the same level of income [5]. Presumably, similar patterns are present in the income-adiposity relationship among high SES women in SSA because existing evidence suggests that highly educated individuals have better diets and are more likely to exercise than the less educated [6]. Examining the effect modification of educational attainment on the association between household wealth status and overweight and obesity in the SSA context informs the identification of at-risk sub-populations, additional mechanistic research and increased specificity in the targeting of obesity prevention interventions at both the individual and ecological level. However, studies investigating how different combinations of household wealth status and educational attainment relate to adult female overweight and obesity, specifically in most low- and middle-income countries (LMICs) in sub-Saharan African countries are non-existent, which provides the rationale for this study.

Although a previous study examined the modification of the effect of household wealth on obesity by educational attainment in two SSA countries, our appraisal suggests several issues [7]. First, this study precluded far-reaching conclusions on the nature of the examined individual and household characteristics in the region because analyses were completed for only two of the 48 SSA countries - Benin and Nigeria [7]. Second, Nigeria, one of the two countries in the study, was classified as a low-income country, although the World Bank classification at the time of the study indicated that Nigeria was a lower-middle-income country, and remains so to date [7]. Third, emerging evidence indicates an increased risk of morbidity and mortality from chronic diseases such as cardiovascular diseases and cancers among pre-obese or overweight individuals, which is a body mass index (BMI) of 25 kg/m^2^ to 29.9 kg/m^2^; however, the interrelationship between SES and pre-obesity was not evaluated [8–10]. Finally, the interpretation of the study findings was solely based on notions of statistical significance [7].

In particular, the authors concluded that educational attainment modifies the association of household wealth with obesity only in middle-income but not in low-income countries based on a statistically significant measure of effect modification less than one [7]. We deem this claim unjustified on the basis of the small number of countries studied and the incorrect classification of Nigeria as a low-income country, which we believe impacted the study conclusions, and the use of statistical significance (*p* < 0.05) as the sole criterion for arriving at the study’s conclusions.

The American Statistical Association (ASA) provided recent guidance stating that researchers should deviate from reaching research study conclusions on the absence or presence of a phenomenon solely on the basis of statistically significant findings [11]. This is because declarations of statistical significance and non-significance is not an indication of the presence or absence of an association or effect in reality, especially when premised on a single study [11]. Therefore, researchers are advised to be modest; use larger samples; control, explore and communicate the uncertainty in their estimates and embrace meta-analytic thinking in providing research guidance [11]. These observed gaps in the literature warrants further study to clearly delineate the interrelationships between SES factors and overweight and obesity within the SSA region to support future research and meta-analytic work.

The objective of this study was to examine whether the positive effect of middle-income and rich household wealth on adult SSA women’s overweight status was modified by the level of education attainment. We hypothesized that the positive effect of household wealth on overweight status would be lower among women with secondary or higher education compared to those with primary or no education across SSA countries. We aimed to fill the existing gaps in the literature using a greater number of SSA countries with varying income status to allow broad generalization to the region. Additionally, in this study, we combined the traditional modeling method of logistic regression analysis with Analysis of Credibility (AnCred), a new analytical technique that allows better communication of uncertainty and provision of richer interpretation of research findings [12].

This study, focused on overweight, defined by World Health Organization (WHO) as BMI ≥ 25 kg/m^2^ [13], because the prevalence of female obesity (BMI ≥ 30 kg/m^2^) at the national-level in most SSA countries is quite low [1]. For this reason, using obesity as an outcome in this study would have been inadequately powered.

## Methods

### Data source

The Demographic and Health Surveys (DHS) are nationally and regionally representative household-based surveys of women aged 15–49 years, individually conducted in various countries [14, 15]. During sampling, countries were divided into survey regions, which are sub-national units that correspond to existing administrative units such as states or provinces. These sub-national units were stratified by urban and rural areas and cluster sampling was carried out in two stages. First, from each region, urban/rural stratified random sampling of enumeration units or clusters were conducted. Next, within each enumeration unit, a random sample of households was selected for inclusion in the survey. Information such as household sociodemographic status and women’s reproductive history were self-reported. Trained personnel collected anthropometric data by measuring and recording participants’ weight using the SECA 874 digital scale to the nearest 0.01 kg and height with the Shorr height board. These anthropometric measurements were recorded in the biomarker questionnaire [16]. Detailed description of the survey methodology and administration is published elsewhere [14]. We were only able to access 34 SSA country datasets from the DHS database and these were identified for potential inclusion in the study analysis.

### Conceptual model

In effect-modification analysis, confounding control is typically reserved for the primary exposure [17]. Therefore, in this study, confounders were only identified for household wealth. Since it has been established that dietary factors and the level of physical activity are more direct individual causal factors for overweight and obesity [18–20], it was hypothesized that these variables mediate the relationship between household wealth and overweight in SSA countries and did not require statistical adjustment. Additionally, it was hypothesized that parity, a discrete variable that lists the number of children ever born by respondents, was a potential confounder in this study as it could be influenced by household wealth. Moreover, the existence of cumulative evidence indicating that first birth is associated with weight gain suggested that parity was related to the study outcome, overweight status [21, 22]. By perusing other studies, additional confounders were identified leading to the assumption that the relationship between household wealth and overweight would be confounded by place of residence (urban or rural residence) [23], age [24], and ethnicity-related factors such cultural practices and political hegemony [25–27]. Education was hypothesized as an effect modifier. These relationships were tested in a statistical model, which is described in detail in the statistical analysis section.

### Data management

For the purposes of this study, participants of interest were women aged 18–49 years. Pregnant women were excluded due to gestational weight changes [28]. Additionally, individuals younger than 18 years were also excluded due to the study’s focus on adult women. Both overweight and obesity status were classified as overweight using the WHO Adult BMI classification of ≥25 kg/m^2^. The outcome – BMI category - was categorized as underweight/normal weight (BMI < 24.9 kg/m^2^) or overweight (BMI ≥ 25 kg/m^2^).

The exposure of interest, household wealth index, is a composite measure of household living standards. It was calculated by the DHS program using principal component analysis based on household ownership of specific assets, including television and bicycles, materials for household construction, and types of water sources and sanitation facilities [29]. The DHS dataset contained wealth index factor scores which were ranked into three wealth index tertiles consisting of poor, middle-income, and rich households [15]. Educational attainment was categorized as “no education”, “primary education”, “secondary education” and “higher education” in the original dataset; however, it was recoded as “none or primary education” and “secondary or higher education” for the study analysis. The breakdown of wealth index and education used in this study was to ensure a sufficient number of participants within each stratum of these variables to support the effect modification analysis.

Potential confounders were age, ethnicity, place of residence and parity. Each country dataset contained the age in years for individual participants and it was treated as a continuous variable in the analysis. The variable which captured ethnic identity was used as a surrogate construct for exposure to cultural and political factors. Ethnic groups varied by country and were used in the analysis in the same form as presented within the DHS dataset. In Nigeria and Zimbabwe, where there were more than 60 ethnic categories with several categories containing fewer than 10 respondents, and eight other countries (Comoros, Liberia, Rwanda, Tanzania, Zimbabwe, Lesotho, Swaziland, Namibia) where ethnicity data were not collected, ethnicity was not adjusted for as a confounder in the analysis. The same categories of place of residence as in the original DHS dataset – “urban” and “rural”- were used in the analysis. Parity was treated as a continuous variable in the analysis.

### Statistical analysis

Statistical analysis was conducted using SAS software version 9.2 [30]. First, preliminary descriptive analysis was used to assess the contents of individual country datasets and to estimate the national level prevalence of overweight. Subsequently, the inter-relationships between BMI, household wealth index, and educational attainment were explored on a country-by-country basis in the 34 countries initially considered for inclusion in this study. These 34 SSA countries were those that had publicly available datasets within the DHS website. Country datasets with less than 10 overweight and underweight/normal weight respondents in any cell within the strata of education and wealth did not undergo further analysis. This was because analysis involving small samples were deemed underpowered to detect the true effect. Additionally, small samples would not maintain the balance of randomization equivalent to the true population of the subgroup and were insufficient to compute the true odds of overweight based on the normal approximation to the binomial distribution [31, 32]. As a result, 12 SSA country datasets were excluded and 22 SSA countries with complete data were included in the single and multiple variable models.

Missing data patterns in the selected 22 datasets were assessed to determine the missing data mechanism that will be adopted (missing completely at random (MCAR), missing at random (MAR) and missing not at random (MNAR) as described by Rubin [33]). While it is impossible to determine the missing data pattern with only the observed data, it has been suggested that an MCAR assumption is not appropriate if varying missing data patterns show differences in the distribution of values across groups [34]. Our exploration of the 22 country datasets (not shown) indicated that the mean values for potential confounders and the effect modifier varied when participants were stratified by missing data status. As such, a MAR assumption was adopted, meaning that it was assumed that missing variables can be predicted by related variables in the dataset [35]. SAS software multiple imputation procedures - PROC MI and PROC MIANALYZE - both rely on the MAR assumption for all analyses. Hence PROC MI was used to create multiple imputed individual country datasets for countries with greater than 10% missing values, using a seed value to ensure replication and adjusting for the sample weights and sample strata by including them in the “class” command similar to the work of Berglund [36]. Fully conditional specification method, the multiple imputation technique applied in this study has been previously described [36]. At the end of the imputation process, multicollinearity of the independent variables was assessed (variance inflation factor < 10).

Next, single and multivariable models were constructed separately for individual countries and by the imputed dataset, accounting for the sample weights and strata. This involved using SAS PROC SURVEYLOGISTIC procedure and the associated “stratum” and “weight” syntax. The first model was a simple logistic regression model for testing the association of individual covariates with the outcome variable, BMI category. The second model was a multiple logistic regression model for an effect modification analysis with educational attainment as the effect modifier, household wealth as the exposure and BMI category as the outcome with adjustment for ethnicity, and where applicable, parity, age, and place of residence. The women sample weights, labeled as “v005” in the DHS dataset, was divided by one million and used to adjust the DHS sample so that it was representative of the population of individual countries [37].

Finally, the parameter estimates and the standard errors from each imputed dataset were combined using the SAS software procedure, PROC MIANALYZE, to generate averaged estimates for individual countries. Standard errors were also adjusted for the complex sample design and for the variability resulting from the multiple imputation process. A measure of multiplicative effect modification was computed for middle income and rich tertiles as a ratio of odds ratios (ORs) across strata of educational attainment with 95% CIs calculated using the estimated standard errors. The results of the effect modification analyses were presented according to the recommendations of Konol and VanderWeele [38].

### Analysis of credibility

The credibility or believability of the study findings at the 95% confidence level were analyzed following the recommendations of the American Statistical Association [11] to use analysis of credibility (AnCred) methods, which have been detailed in several publications [12, 39, 40].

In summary, analysis of AnCred methods subjects claims of statistical significance and non-significance to a fair-minded challenge by computing critical prior intervals (CPI), which are a range of prior values, which when combined with the study evidence, produces posterior values that include or exclude no effect [40]. The computed CPI is then compared with effect sizes from prior studies, allowing statistical significant and nonsignificant findings to be deemed credible if those prior studies support values that fall outside the computed CPI [40]. CPIs for statistically significant findings are called Skepticism CPIs and for non-statistically significant findings Advocacy CPIs. The computed limits are known as Skepticism Limits (SL) or Advocacy Limits (AL).

In AnCred methods, the value of novel findings is judged using the concept of intrinsic credibility [40]. Intrinsic credibility in reference to a study finding implies that it provides sufficient evidence of an effect even in the absence of prior studies [40]. Unprecedented findings, which are statistically significant, can be considered intrinsically credible if their most probable value (point estimate) fall outside their SL CPI, that is, their most probable estimate does not correspond to effect sizes that when combined with existing findings includes no effect [40]. Unprecedented non-statistically significant findings with point estimates that support the study hypothesis also follow similar criteria in assessment of intrinsic credibility. For unprecedented non-statistically significant findings contrary to the study hypothesis, they are considered intrinsically credible if the effect sizes supported by their AL are in the opposite direction from those supported by the study hypothesis [40].

Therefore, using AnCred methods, two important questions could be answered: (i) Does the measure of effect modification make a strong case for or against the study hypothesis in the light of prior evidence? (ii) Does the measure of effect modification make a strong case for or against the study hypothesis on its own merit? However, since comparable prior evidence does not exist for the countries included in this study, only the second question was answered by AnCred methods. In this study, the substantive hypothesis was that the measures of effect modification in the middle and rich tertiles were less than one. Therefore, the skepticism CPI is (SL, 1/SL), and the advocacy CPI is (AL, 1) (See Additional file 1 for formulae and derivation of critical prior intervals).

## Results

### Participants

Table 1 lists the 22 country surveys that were included in this study, the original DHS sample size, complete and missing BMI cases, excluded observations and reasons for exclusions. The percentage of missing BMI data is the ratio of the missing BMI observations to the sum of the complete and missing BMI cases. The proportion of missing BMI cases ranged from 0.8 to 68.6%. All included surveys were the most recent DHS surveys available for public use at the time of the study and were conducted prior to 2005. The presentation of results in Table 1 and subsequent tables were grouped by country income status based on the World Bank classification of countries for the year of the survey [41].

**Table 1:** Description of Included Surveys and Overall Overweight Prevalence in 34 SSA Countries

Country and Survey Year	Total DHS sample /complete BMI cases	Missing BMI data (% of total BMI sample)	Exclusions (Numbers)	Overweight Prevalence and 95% CI
*Low Income Countries*				
1. Benin 2011–2012	16,599/12,811	372 (2.8)	Adolescents (1869), Pregnant women (1547)	28.9 (27.9–30.0)
2. Chad 2014–2015	17,719/8367	4655 (35.7)	Adolescent (2411), Pregnant women (2286)	12.6 (11.6–13.6)
3. Comoros 2012	5329/4102	122 (2.9)	Adolescents (779), Pregnant women (326)	41.0 (38.7–43.3)
4. Congo DRC 2013–2014	18,827/7031	7168 (50.5)	Adolescents (2376), Pregnant women (2252)	17.3 (15.3–19.3)
5. Gambia 2013	10,233/3603	4457 (55.3)	Adolescents (1374), Pregnant women (799)	24.9 (23.2–26.6)
6. Liberia 2013	9239/3654	3648 (50.0)	Adolescents (1192), Pregnant women (745)	29.7 (27.0–32.5)
7. Malawi 2015–2016	24,562/6443	13,264 (67.3)	Adolescents (3170), Pregnant women (1685)	23.1 (21.7–24.4)
8. Rwanda 2014–2015	13,497/5361	5450 (50.4)	Adolescents (1743), Pregnant women (943)	22.5 (21.2–23.8)
9. Sierra Leone 2013	16,658/6269	6653 (51.5)	Adolescents (2416), Pregnant women (1.320)	20.3 (18.5–22.0)
10. Tanzania 2015–2016	13,266/10,369	86 (0.8)	Adolescents (1749), Pregnant women (1062)	31.4 (29.9–33.0)
11. Togo 2013–2014	9480/3875	3796 (49.5)	Adolescents (1019), Pregnant women (790)	33.3 (31.5–35.2)
12. Zimbabwe 2015	9955/7758	251 (3.1)	Adolescent (1348), Pregnant women (569)	38.9 (37.3–40.4)
*Lower-middle Income Countries*				
13. Cameroon 2011	15,426/6091	5804 (48.8)	Adolescents (2142), Pregnant women (1389)	35.1 (33.5–36.6)
14. Congo 2011–2012	10,819/4414	4072 (48.0)	Adolescents (1326), Pregnant women (1007)	29.3 (26.9–31.7)
15. Ghana 2014	9396/3845	3785 (49.6)	Adolescents (1101), Pregnant women (665)	44.3 (42.0–46.6)
16. Kenya 2014	31,079/11,762	13,581 (53.6)	Adolescents (3769), Pregnant women (1967)	35.8 (34.4–37.2)
17. Lesotho 2014	6621/2764	2656 (49.0)	Adolescents (949), Pregnant women (252)	49.3 (47.0–51.7)
18. Nigeria 2013	38,948/29,304	442 (1.49)	Adolescents (4946), Pregnant women (4256)	27.6 (26.6–28.6)
19. Swaziland 2006–2007	4987/3855	110 (2.8)	Adolescents (782), Pregnant women (240)	56.6 (54.8–58.5)
20. Zambia 2013–2014	16,411/12,809	137 (1.1)	Adolescent (2168), Pregnant women (1297)	25.1 (24.0–26.1)
*Upper-middle Income Countries*				
21. Gabon 2012	8422/4265	2251 (34.5)	Adolescents (1121), Pregnant women (785)	48.7 (46.2–51.3)
22. Namibia 2013	10,018/4407	3994 (47.5)	Adolescents (1072), Pregnant women (545)	35.2 (33.4–37.0)

### Descriptive data

Besides the survey description, Table 1 also shows the national level prevalence of overweight among women aged 18–49 years for all 22 countries. Chad had the lowest overweight prevalence at 12.6% while the highest overweight prevalence was observed in Swaziland at 56.6%.

Table 2 presents the number and proportion of overweight women within strata of education attainment and wealth tertiles for all 22 countries considered in the study. In the secondary or higher education stratum, the number and proportion of overweight women increased with rising household wealth. Whereas, in the no or primary education stratum, it varied within individual wealth tertiles.

**Table 2:** Description of the Numbers and Proportion of Overweight Women within Strata of Household Wealth and Educational Attainment in 22 SSA Countries

		Poorest		Middle		Rich	
Country	Educational Attainment	Overweight (N, %)	Underweight or normal weight (N, %)	Overweight (N, %)	Underweight or normal weight (N, %)	Overweight (N, %)	Underweight or normal weight (N, %)
*Low- Income Countries*							
1. Benin 2011–12	None/Primary	720 (17.1)	3453 (82.9)	1038 (27.6)	2799 (72.4)	1074 (43.1)	1524 (56.9)
	Secondary/Higher	20 (14.1)	130 (85.9)	81 (17.4)	385 (82.6)	565 (36.2)	1022 (63.8)
2. Chad 2014–2015	No/nePrimary	226 (8.3)	2476 (91.7)	190 (8.2)	2347 (91.8)	388 (19.3)	1763 (80.7)
	Secondary/Higher	14 (10.0)	126 (90.0)	26 (10.2)	155 (89.8)	167 (26.2)	489 (73.8)
3. Comoros 2012	No/Primary	414 (38.8)	570 (61.2)	325 (46.7)	364 (53.3)	220 (53.7)	183 (46.3)
	Secondary/Higher	96 (20.9)	287 (79.1)	247 (38.0)	429 (62.0)	413 (42.2)	541 (57.8)
4. Congo DRC 2013–2014	None/Primary	157 (8.7)	1727 (91.3)	176 (11.3)	1436 (88.7)	145 (30.2)	464 (69.7)
	Secondary/Higher	23 (3.7)	455 (96.3)	72 (8.6)	701 (91.4)	497 (31.2)	1178 (68.8)
5. Gambia 2013	None/Primary	189 (18.5)	825 (81.5)	200 (23.7)	678 (76.3)	181 (36.2)	281 (63.8)
	Secondary/Higher	29 (11.0)	223 (89.0)	58 (22.2)	235 (77.8)	219 (29.2)	485 (70.8)
6. Liberia 2013	None/Primary	223 (19.9)	960 (80.1)	230 (22.3)	707 (77.7)	234 (41.4)	345 (58.6)
	Secondary/Higher	20 (22.8)	80 (77.2)	46 (19.7)	196 (80.3)	224 (34.5)	389 (65.5)
7. Malawi 2015–2016	None/Primary	254 (13.4)	1722 (86.6)	356 (20.4)	1326 (79.6)	355 (37.2)	569 (62.8)
	Secondary/Higher	24 (10.5)	172 (89.5)	82 (21.1)	349 (78.9)	488 (38.1)	746 (61.9)
8. Rwanda 2014–2015	None/Primary	231 (13.3)	1483 (86.7)	275 (18.7)	1172 (81.3)	359 (36.6)	602 (63.4)
	Secondary/Higher	15 (11.5)	104 (88.5)	59 (20.4)	219 (79.6)	324 (37.7)	518 (62.3)
9. Sierra Leone 2013	None/Primary	256 (12.7)	1656 (87.3)	328 (17.8)	1369 (82.2)	341 (35.2)	630 (64.8)
	Secondary/Higher	18 (10.5)	172 (89.5)	57 (14.8)	330 (85.2)	310 (29.4)	802 (70.6)
10. Tanzania 2015–2016	None/Primary	521 (15.6)	2730 (84.4)	788 (29.2)	1941 (70.8)	873 (51.2)	823 (48.8)
	Secondary/Higher	37 (14.2)	177 (85.8)	211 (23.5)	520 (76.5)	813 (48.0)	935 (52.0)
11. Togo 2013–2014	None/Primary	158 (15.8)	992 (84.2)	273 (29.5)	671 (70.5)	326 (53.8)	282 (46.2)
	Secondary/Higher	20 (13.5)	143 (86.5)	81 (25.6)	242 (74.4)	294 (43.5)	393 (56.5)
12. Zimbabwe 2015	None/Primary	334 (25.8)	949 (74.2)	232 (41.9)	299 (58.1)	94 (54.3)	81 (45.7)
	Secondary/Higher	371 (27.4)	989 (72.6)	873 (41.3)	1201 (58.7)	1253 (53.0)	1111 (47.0)
*Lower-middle Income Countries*							
13. Cameroon 2011	None/Primary	302 (15.7)	1424 (84.3)	418 (35.9)	761 (64.1)	251 (52.1)	235 (47.9)
	Secondary/Higher	80 (26.9)	207 (73.1)	306 (36.6)	560 (63.4)	750 (49.5)	797 (50.5)
14. Congo 2011–2012	None/Primary	99 (10.4)	870 (89.6)	110 (20.3)	550 (80.0)	73 (32.8)	162 (67.2)
	Secondary/Higher	79 (17.2)	410 (82.8)	164 (19.8)	697 (80.2)	456 (39.1)	744 (60.9)
15. Ghana 2014	Nome/Primary	169 (18.1)	759 (81.9)	228 (40.8)	311 (59.2)	150 (65.1)	84 (34.9)
	Secondary/Higher	61 (20.6)	280 (79.4)	305 (40.7)	456 (59.3)	611 (60.8)	431 (39.2)
16. Kenya 2014	None/Primary	509 (16.3)	2891 (83.7)	875 (33.3)	1711 (66.7)	752 (52.3)	695 (47.7)
	Secondary/Higher	102 (19.5)	424 (80.5)	409 (28.8)	1010 (71.2)	1188 (49.6)	1196 (50.4)
17. Lesotho 2014	None/Primary	234 (36.1)	436 (63.9)	192 (52.9)	168 (46.1)	108 (62.1)	64 (37.9)
	Secondary/Higher	99 (34.8)	182 (65.2)	290 (51.2)	281 (48.8)	423 (56.5)	287 (43.5)
18. Nigeria 2013	None/Primary	1192 (13.0)	7571 (87.0)	1489 (27.1)	3901 (72.9)	846 (45.1)	1038 (54.9)
	Secondary/Higher	157 (15.6)	835 (84.4)	1096 (24.6)	3282 (75.4)	3368 (42.6)	4529 (57.4)
19. Swaziland 2006–2007	None/Primary	391 (49.0)	413 (51.0)	295 (60.8)	182 (39.2)	166 (69.7)	83 (30.3)
	Secondary/Higher	225 (46.7)	265 (53.3)	447 (56.2)	366 (43.8)	646 (63.4)	376 (36.6)
20. Zambia 2013–2014	None/Primary	396 (11.4)	3163 (88.6)	567 (22.6)	2036 (77.4)	436 (43.7)	545 (56.3)
	Secondary/Higher	69 (10.1)	628 (89.9)	354 (19.7)	1326 (80.3)	1242 (38.6)	2040 (61.4)
*Upper-middle Income Countries*							
21. Gabon 2012	None/Primary	266 (28.6)	652 (71.4)	257 (55.7)	261 (44.3)	160 (63.1)	104 (36.9)
	Secondary/Higher Education	171 (34.8)	354 (65.2)	374 (43.8)	529 (56.2)	562 (51.6)	575 (48.4)
22. Namibia 2013	None/Primary Education	111 (18.7)	425 (81.3)	139 (42.7)	184 (57.3)	55 (68.2)	31 (31.8)
	Secondary/Higher Education	131 (18.0)	519 (82.0)	357 (37.0)	582 (63.0)	550 (47.0)	529 (53.0)

### Main findings

#### Effect modification analysis

Table 3 and its supplement (Expanded Table 3) shows the results of the effect modification analysis and AnCred after adjustment for age, parity, place of residence and ethnicity in 22 countries. Point estimates of the measure of effect modification, the ratio of OR, below one means that the estimated effect of household wealth in relation to overweight was smaller among those with secondary or higher education compared to those with no or primary education. Alternatively, point estimates of effect modification greater than one means that the estimated effect of household wealth in relation to overweight was larger among those with secondary or higher education compared to those with no or primary education. The majority of countries under evaluation (18 of the 22 countries) had point estimates for the measure of effect modification below one in either the middle-income or rich tertile or both. Specifically, seven (Benin, Chad, Liberia, Sierra Leone, Tanzania, Togo, and Zimbabwe) of the 12 low-income countries had measures of effect modification that were below one for both the middle and rich tertiles while the measure of effect modification for Rwanda was only less than one in the rich tertile. All 10 lower-middle and upper–middle income countries considered in this study had point estimates that were consistently below one.

**Table 3:** Modification of the Effect of Household Wealth on Overweight by Educational Attainment in 22 SSA Countries

		Poorest	Middle	Rich	OR (95% CI) for middle-income household wealth tertile	OR (95% CI) for rich household wealth tertile	AnCred for middle tertile		AnCred for rich tertitle	
Country	Educational Attainment	OR (95% CI)	OR (95% CI)	OR (95% CI)			Scepticism/Advocacy limit (OR)	Intrinsically credible if no prior evidence	Scepticism/Advocacy limit (OR)	Intrinsically credible if no prior evidence
*Low- Income Countries*										
1. Benin 2011–12	None/Primary	1.00	1.65 (1.44–1.89)	2.74 (2.34–3.21)	1.65 (1.44–1.89)	2.74 (2.34–3.21)				
	Secondary/Higher	1.34 (0.77–2.33)	1.42 (1.07–1.90)	2.39 (1.98–2.89)	1.06 (0.58–1.95)	1.78 (1.02–3.11)				
	*Measure of effect modification on multiplicative scale: ratio of ORs (95% CIs)*				0.64 (0.35–1.17)	0.65 (0.37–1.15)	AL = 0.02	no	AL = 0.02	no
2. Chad 2014–15	None/Primary	1.00	1.04 (0.80–1.36)	1.63 (1.25–2.12)	1.04 (0.80–1.36)	1.63 (1.25–2.12)				
	Secondary/Higher	1.89 (0.98–3.64)	1.85 (0.99–3.49)	2.22 (1.59–3.10)	0.98 (0.42–2.26)	1.17 (0.62–2.24)				
	*Measure of effect modification on multiplicative scale: ratio of ORs (95% CIs)*				0.94 (0.40–2.21)	0.72 (0.37–1.42)	AL = 0.78	no	AL = 0.19	no
3. Comoros 2012	None/Primary	1.00	1.37 (1.08–1.75)	1.76 (1.30–2.37)	1.37 (1.08–1.75)	1.76 (1.30–2.37)				
	Secondary/Higher	0.68 (0.47–0.99)	1.41 (1.07–1.86)	1.45 (1.11–1.90)	2.07 (1.42–3.02)	2.13 (1.51–3.02)				
	*Measure of effect modification on multiplicative scale: ratio of ORs (95% CIs)*				1.51 (0.98–2.32)	1.22 (0.76–1.93)	AL > 1000	no	AL = 2.5	no
4. Congo DRC 2013–14	None/Primary	1.00	1.12 (0.84–1.49)	3.01 (2.16–4.18)	1.12 (0.84–1.49)	3.01 (2.16–4.18)				
	Secondary/Higher	0.79 (0.48–1.32)	1.36 (0.92–2.03)	4.59 (3.37–6.25)	1.72 (0.96–3.07)	5.77 (3.35–9.95)				
	*Measure of effect modification on multiplicative scale: ratio of ORs (95% CIs)*				1.53 (0.80–2.93)	1.92 (1.08–3.40)	AL = 19.93	no	SL = 2.9	yes
5. Gambia 2013	None/Primary	1.00	1.32 (0.99–1.74)	1.86 (1.29–2.69)	1.32 (0.99–1.74)	1.86 (1.29–2.69)				
	Secondary/Higher	0.99 (0.64–1.55)	1.61 (1.10–2.37)	1.99 (1.38–2.89)	1.62 (0.98–2.67)	2.01 (1.31–3.07)				
	*Measure of effect modification on multiplicative scale: ratio of ORs (95% CIs)*				1.23 (0.71–2.15)	1.08 (0.65–1.79)	AL = 2.69	yes	none available	yes
6. Liberia	None/Primary	1.00	1.18 (0.92–1.53)	3.22 (2.11–4.92)	1.18 (0.92–1.53)	3.22 (2.11–4.92)				
	Secondary/Higher	1.29 (0.71–2.35)	1.47 (0.97–2.25)	2.85 (1.64–4.95)	1.14 (0.61–2.11)	2.85 (1.64–4.95)				
	*Measure of effect modification on multiplicative scale: ratio of ORs (95% CIs)*				0.96 (0.51–1.83)	0.89 (0.46–1.72)	AL = 0.87	no	AL = 0.62	no
7. Malawi	None/Primary	1.00	1.37 (1.13–1.67)	2.94 (2.35–3.67)	1.37 (1.13–1.67)	2.94 (2.35–3.67)				z
	Secondary/Higher	1.06 (0.63–1.78)	1.76 (1.29–2.38)	3.28 (2.57–4.20)	1.66 (0.91–3.05)	3.11 (1.96–4.92)				
	*Measure of effect modification on multiplicative scale: ratio of ORs (95% CIs)*				1.21 (0.64–2.30)	1.06 (0.64–1.75)	none available	yes	none available	yes
8. Rwanda	None/Primary	1.00	1.39 (1.16–1.66)	3.02 (2.47–3.68)	1.39 (1.16–1.66)	3.02 (2.47–3.68)				
	Secondary/Higher	1.21 (0.66–2.23)	1.85 (1.30–2.63)	3.53 (2.87–4.35)	1.53 (0.79–2.97)	2.92 (1.58–5.40)				
	*Measure of effect modification on multiplicative scale: ratio of ORs (95% CIs)*				1.10 (0.56–2.16)	0.97 (0.51–1.83)	none available	yes	AL = 0.87	no
9. Sierra Leone 2013	None/Primary	1.00	1.36 (1.07–1.71)	2.67 (1.99–3.60)	1.36 (1.07–1.71)	2.67 (1.99–3.60)				
	Secondary/Higher	1.23 (0.74–2.06)	1.57 (1.07–2.30)	2.49 (1.79–3.47)	1.27 (0.71–2.28)	2.02 (1.20–3.42)				
	*Measure of effect modification on multiplicative scale: ratio of ORs (95% CIs)*				0.94 (0.50–1.77)	0.76 (0.45–1.27)	AL = 0.78	no	AL = 0.21	no
10. Tanzania 2015–16	None/Primary	1.00	2.13 (1.82–2.50)	5.46 (1.82–2.50)	2.13 (1.82–2.50)	5.46 (4.29–6.95)				
	Secondary/Higher	1.67 (1.04–2.67)	2.68 (2.04–3.52)	6.00 (4.78–7.53)	1.61 (0.95–2.72)	3.60 (2.19–5.89)				
	*Measure of effect modification on multiplicative scale: ratio of ORs (95% CIs)*				0.75 (0.44–1.30)	0.66 (0.40–1.08)	AL = 0.22	no	AL = 0.003	no
11. Togo 2013–14	None/Primary	1.00	1.70 (1.25–2.30)	3.84 (2.42–6.09)	1.70 (1.25–2.30)	3.84 (2.42–6.09)				
	Secondary/Higher	1.24 (0.72–2.15)	1.93 (1.29–2.87)	4.00 (2.54–6.30)	1.55 (0.82–2.93)	3.22 (1.73–6.01)				
	*Measure of effect modification on multiplicative scale: ratio of ORs (95% CIs)*				0.91 (0.49–1.70)	0.84 (0.47–1.50)	AL = 0.69	no	AL = 0.46	no
12. Zimbabwe 2015	None/Primary	1.00	2.13 (1.69–2.68)	3.52 (2.34–5.29)	2.13 (1.69–2.68)	3.52 (2.34–5.29)				
	Secondary/Higher	1.37 (1.14–1.65)	2.56 (2.07–3.16)	4.18 (3.24–5.40)	1.87 (1.53–2.28)	3.05 (2.37–3.94)				
	*Measure of effect modification on multiplicative scale: ratio of ORs (95% CIs)*				0.88 (0.67–1.16)	0.87 (0.58–1.31)	AL = 0.53	no	AL = 0.54	no
*Lower-Middle Income Countries*										
13. Cameroon 2011	None/Primary	1.00	2.03 (1.58–2.60)	3.80 (2.80–5.17)	2.03 (1.58–2.60)	3.80 (2.80–5.17)				
	Secondary/Higher	1.58 (1.09–2.29)	2.22 (1.59–3.11)	3.76 (2.90–4.89)	1.41 (0.97–2.05)	2.38 (1.64–3.47)				
	*Measure of effect modification on multiplicative scale: ratio of ORs (95% CIs)*				0.69 (0.48–1.00)	0.63 (0.40–0.98)	AL < 0.01	no	SL = 0.23	no
14. Congo 2011–12	No/Primary Education	1.00	1.60 (1.12–2.28)	3.15 (2.05–4.84)	1.60 (1.12–2.28)	3.15 (2.05–4.84)				
	Secondary/Higher	1.53 (1.08–2.17)	1.85 (1.29–2.66)	4.25 (3.04–5.95)	1.21 (0.79–1.85)	2.78 (1.89–4.09)				
	*Measure of effect modification on multiplicative scale: ratio of ORs (95% CIs)*				0.76 (0.44–1.32)	0.88 (0.54–1.44)	AL = 0.24	no	Al = 0.58	no
15. Ghana 2014	None/Primary	1.00	2.69 (1.95–3.73)	7.10 (4.60–11.0)	2.69 (1.95–3.73)	7.10 (4.60–11.0)				
	Secondary/Higher	1.42 (0.93–2.18)	3.31 (2.33–4.69)	7.74 (5.41–11.08)	2.33 (1.66–3.27)	5.45 (3.80–7.81)				
	*Measure of effect modification on multiplicative scale: ratio of ORs (95% CIs)*				0.86 (0.55–1.35)	0.77 (0.45–1.30)	AL = 0.51	no	AL = 0.24	no
16. Kenya 2014	None/Primary	1.00	1.98 (1.72–2.28)	4.16 (3.46–5.01)	1.98 (1.72–2.28)	4.16 (3.46–5.01)				
	Secondary/Higher	1.28 (0.98–1.66)	2.00 (1.68–2.37)	4.13 (3.49–4.89)	1.56 (1.18–2.07)	3.24 (2.49–4.21)				
	*Measure of effect modification on multiplicative scale: ratio of ORs (95% CIs)*				0.79 (0.58–1.07)	0.77 (0.59–1.02)	AL = 0.09	no	AL < 0.01	no
17. Lesotho	None/Primary	1.00	1.98 (1.51–2.60)	2.90 (1.93–4.36)	1.98 (1.51–2.60)	2.90 (1.93–4.36)				
	Secondary/Higher	1.46 (1.08–1.99)	2.79 (2.18–3.57)	3.81 (2.88–5.03)	1.91 (1.39–2.62)	2.60 (1.80–3.77)				
	*Measure of effect modification on multiplicative scale: ratio of ORs (95% CIs)*				0.96 (0.63–1.48)	0.90 (0.56–1.44)	AL = 0.89	no	AL = 0.64	no
18. Nigeria	None/Primary	1.00	2.28 (1.97–2.66)	4.50 (3.75–5.40)	2.28 (1.97–2.66)	4.50 (3.75–5.40)				
	Secondary/Higher	2.01 (1.57–2.58)	3.20 (2.75–3.72)	6.15 (5.28–7.15)	1.59 (1.25–2.03)	3.06 (2.40–3.91)				
	*Measure of effect modification on multiplicative scale: ratio of ORs (95% CIs)*				0.70 (0.53–0.92)	0.68 (0.51–0.90)	SL = 0.72	yes	SL = 0.74	yes
19. Swaziland	None/Primary	1.00	1.94 (1.47–2.56)	4.01 (2.68–6.01)	1.94 (1.47–2.56)	4.01 (2.68–6.01)				
	Secondary/Higher	1.54 (1.21–1.97)	2.48 (1.92–3.20)	3.29 (2.58–4.20)	1.61 (1.18–2.18)	2.13 (1.62–2.81)				
	*Measure of effect modification on multiplicative scale: ratio of ORs (95% CIs)*				0.83 (0.58–1.18)	0.53 (0.34–0.82)	AL = 0.35	no	SL = 0.66	yes
20. Zambia	None/Primary	1.00	2.02 (1.67–2.43)	5.27 (4.18–6.63)	2.02 (1.67–2.43)	5.27 (4.18–6.63)				
	Secondary/Higher	1.37 (1.00–1.88)	2.66 (2.18–3.25)	6.14 (4.97–7.60)	1.94 (1.43–2.62)	4.48 (3.25–6.17)				
	*Measure of effect modification on multiplicative scale: ratio of ORs (95% CIs)*				0.96 (0.67–1.38)	0.85 (0.59–1.22)	AL = 0.85	no	AL = 0.44	no
*Upper-Middle Income Countries*										
21. Gabon	None/Primary	1.00	2.88 (1.96–4.23)	3.98 (2.47–6.41)	2.88 (1.96–4.23)	3.98 (2.47–6.41)				
	Secondary/Higher	1.60 (1.12–2.28)	3.02 (1.99–4.59)	3.59 (2.41–5.34)	1.89 (1.27–2.81)	2.25 (1.55–3.26)				
	*Measure of effect modification on multiplicative scale: ratio of ORs (95% CIs)*				0.66 (0.42–1.04)	0.57 (0.35–0.92)	AL < 0.01	no	SL = 0.45	no
22. Namibia	None/Primary	1.00	2.68 (1.91–3.77)	5.50 (3.05–9.91)	2.68 (1.91–3.77)	5.50 (3.05–9.91)				
	Secondary/Higher	1.51 (1.06–2.14)	3.76 (2.71–5.21)	5.94 (4.15–8.50)	2.49 (1.94–3.21)	3.94 (2.93–5.30)				
	*Measure of effect modification on multiplicative scale: ratio of ORs (95% CIs)*				0.93 (0.62–1.38)	0.72 (0.38–1.36)	AL = 0.72	no	AL = 0.17	no

In the middle tertile of countries with point estimates below one, the measure of effect modification ranged from 0.64 to 0.96. Whereas, in the rich tertile of countries with point estimates below one, it ranged from 0.53 to 0.97. An examination of the middle tertile of countries with point estimates of the measures of effect modification above one revealed that the measure of effect modification ranged from 1.10 to 1.53 for those with secondary education; and in the rich tertile, it ranged from 1.06 to 1.92.

#### Intrinsic credibility

Overall, the results from 18 countries provided some support for the study’s hypothesis in at least one wealth tertile. Statistically significant measures of effect modification supporting the study hypothesis were found in three lower-middle income countries and one upper-middle income country. These were Cameroon – rich tertile (0.63; 95% CI, 0.40–0.98), Nigeria – middle tertile (0.70; 0.53–0.92) and rich tertile (0.68; 0.51–0.90), Swaziland – rich tertile (0.53; 0.34–0.82), and Gabon – rich tertile (0.57; 0.35–0.92).

Assessment of the intrinsic credibility (Table 3 & Expanded Table 3) of the measures of effect modification for Nigeria and Swaziland indicated that they were intrinsically credible, which means that the study findings provided evidence of a genuine effect. Conversely, the measure of effect modification for Cameroon and Gabon lacked intrinsic credibility, meaning that they require additional supporting studies with similar effect sizes to make the case for a genuine effect.

A statistically significant measure of effect modification contradicting the study hypothesis was found in one low-income country. This was in the Democratic Republic of Congo’s (DRC) rich tertile, where women of higher educational attainment had an increased likelihood of overweight (1.92, 95% CI, 1.08–3.40). This result was not considered to be intrinsically credible because the point estimate fell within the computed skepticism limit (0.34, 2.90).

Non-statistically significant measures of effect modification were found in one or both tertiles of all 12 low-income countries, one or both tertiles of seven middle-income countries, and one or both tertiles of all two upper-middle-income countries. The majority of these non-significant findings lacked intrinsic credibility, meaning that they required further supporting evidence to strengthen their claim of a null effect. These included one or both tertiles of eight low-income countries (Benin, Chad, Liberia, Rwanda, Sierra Leone, Tanzania, Togo, Zimbabwe), one or both tertiles of six middle income countries (Congo, Ghana, Kenya, Lesotho, Swaziland, Zambia), and one or both tertiles of all two upper-middle-income countries (Ghana and Namibia). Therefore, on the basis of their central estimate, which is below one, we concluded that the measures of effect modification from all 16 countries provide some support for the study hypothesis.

Non-statistically significant measures of effect modification with intrinsic credibility were found in one or both tertiles of four low-income countries (Comoros, Gambia, Malawi, Rwanda), and one or both tertiles of two upper-middle income countries (Cameroon and Nigeria). The ALs from these countries did not offer effect sizes in the direction of the study hypothesis that could be used to oppose the claim of non-significance.

Advocacy limits that were too large in the direction of a positive effect or too small in the direction of a negative effect (Comoros’s middle tertile, DRC middle tertile, Tanzania rich tertile, Cameroon middle tertile, Kenya rich tertile, Gabon middle tertile) provided no support for the effect in either direction since they did not provide useful advocacy limits.

## Discussion

In this 22 SSA countries study, the effect modification of education on the association between household wealth and overweight, among adult women, 18–49 years, was examined and AnCred methods were used to analyze the intrinsic credibility of the study findings. Assessment of intrinsic credibility highlighted the evidential weight of the computed measures of effect modification.

Overall, the results of the intrinsic credibility analysis indicated that there was more support for this study’s hypothesis, that the positive effect of household wealth on overweight status was lower among women with secondary or higher education compared to those with primary or no education, than against it. Eighteen (82%) of the 22 SSA countries had measures of effect modification which provided some degree of support for the study’s hypothesis. Intrinsically credible and statistically significant findings in support of the study’s hypothesis were found in Nigeria and Swaziland.

Further support for this study’s hypothesis was found among the non-significant findings from 16 countries, which were not intrinsically credible. That is, they had a weak claim of non-significance based on the AnCred analysis, and because the central estimate of these non-significant findings was below one, it was concluded that they provided some measure of support for the study’s hypothesis.

Collectively, the findings of this work suggest that women with lower levels of educational attainment in the middle-income and rich wealth strata may have greater vulnerability to the effect of household wealth in its relation to overweight in both low- and middle-income SSA countries. This implies that targeting and successfully addressing the adiposity burden and risk factors among low literacy middle-income or rich adult women could potentially lead to declines in overweight and obesity and associated non-communicable diseases (NCDs) in the region. However, the accumulation of comparable evidence using prior and new data will provide the evidential weight for the study’s findings and must be the first step before accurate research, policy and program recommendations can be dispensed. This study sets the tone for further work by computing skepticism limits and advocacy limits that prior evidence must exclude in order for the study’s findings to be considered credible.

Unfortunately, comparable prior studies do not currently exist. The methods applied in this study differ from prior studies in the construction and analysis of household wealth index. In our analysis, wealth index factor scores were ranked into three tertiles to ensure that the analysis was adequately powered. Whereas, in previous work [7], wealth index was used in its original format, that is as quintiles, and was included as a continuous variable in the multivariate analysis, which led to differing results and interpretations.

It is recommended that subsequent studies use this study’s methodology because wealth index remains a meaningful variable in the present construction as tertiles (the poorest third of the population serves as a comparison group to those in the middle third and in the richest third), while providing adequate sample size for subgroup analyses. Analysis of new data is also encouraged because it is possible that this study captured an emerging phenomenon that will only be supported by more recent data.

Several SSA countries are experiencing nutrition and technological transitions [42], within cultural contexts with a preference for larger body sizes [3, 43, 44] and negative perception of structured physical activity among women and girls [45, 46], which may set the stage for a future epidemic of obesity-related NCDs. Therefore, this calls for urgency in the introduction of new studies to clearly delineate context-appropriate adiposity determinants and intervention mixes. It is evident though that more buy-in from policymakers are needed as African countries have the least amount of funding for the primary prevention of NCDs and NCDs research compared to other countries outside the region [47]. Hopefully, the findings of this study will encourage more research funding to provide the cumulative evidence to support efficiency and effectiveness in the targeting of primary prevention activities.

The findings of this work should be viewed in the light of several limitations. First, the cross-sectional nature of this study implies that the temporal sequence of observed associations in this study is unclear, and the causal nature of the modifying effect of education cannot be assessed. Hence, the positive relationship between household wealth and overweight within strata of educational attainment is correlational. Second, the categorization of educational attainment into two groups of no/primary education, and secondary or higher education may mask heterogeneity that could exist within groups such as college educated individuals. However, this was deemed necessary because of the small numbers of individuals in the college educated category and to ensure sufficient sample sizes for the effect modification analysis. Third, this study only computed multiplicative measures of effect modification and not additive measures of effect modification due to challenges of computing their confidence intervals. While it is recommended that investigators compute both measures of effect modification on both scales [38], in practice, methods for computation of additive interaction require further development in standard software such as SAS. Fourth, although the AnCred methods placed stringent requirements for measures of effect modification to be considered intrinsically credible, the lack of comparable prior evidence weakens the conclusions that can be drawn from the findings of this study. Finally, despite being the most comprehensive study on this topic in the region, the analyses in this study only encompassed 22 out of the 48 SSA countries and may not be truly representative of regional dynamics.

## Conclusions

This study examined the multiplicative effect modification of educational attainment on the positive relationship between household wealth index and overweight in 22 low- and middle-income SSA countries with complete data and found that the effect of household wealth on overweight was greater among adult women with lower levels of educational attainment than those higher levels of educational attainment. The application of AnCred methods enabled a transparent quantitative assessment of the genuineness of the computed measures of effect modification. Additional research to test the credibility of these findings and further delineate the characteristics of educational attainment that decrease vulnerability to overweight in adult women is required.

## Supplementary information

**Additional file 1.**

## Data Availability

The datasets generated and/or analyzed during the current study are publicly available in the DHS program repository, [https://dhsprogram.com/].

## Abbreviations

AL: Advocacy limits
AnCred: Analysis of credibility
ASA: American Statistical Association
BMI: Body mass index
CPI: ritical prior intervals
DRC: Democratic Republic of Congo
DHS: Demographic and Health Survey
LUMC: Lower- and upper-middle income countries
NCD: Noncommunicable diseases
SES: Socioeconomic status
SL: Skepticism limits
SSA: sub-Saharan Africa
WHO: World Health Organization
