# Supplementary material for: Is the positive association between middle-income and rich household wealth and adult sub-Saharan African women’s overweight status modified by the level of education attainment? A cross-sectional study of 22 countries
Source: BMC Public Health. 2020 Jun 25;20:996. doi: 10.1186/s12889-020-08956-3 (PMC7318408; doi:10.1186/s12889-020-08956-3)
Supplement: Supplementary file 1 — Additional file 1. [file 12889_2020_8956_MOESM1_ESM.docx]

**Statistical Appendix**

Is the positive association between middle income and rich household wealth and adult sub-Saharan African women’s overweight status modified by the level of education attainment? A cross-sectional study of 22 countries

Ifeoma D. Ozodiegwu, DrPH^1^, Henry V. Doctor, PhD^2^, Megan Quinn, DrPH^1^, Laina D. Mercer, PhD^3^, Ogbebor Enaholo Omoike, MD ^1^, Hadii M. Mamudu, PhD^4^

^1^Department of Biostatistics and Epidemiology, East Tennessee State University, Johnson City, Tennessee, United States of America

^2^Department of Science, Information, and Dissemination, World Health Organization, Regional Office for the Eastern Mediterranean, Cairo, Egypt

^3^Institute for Disease Modeling, Bellevue, Washington, United States of America (Current address: PATH, Seattle, Washington, United States of America)

^4^Department of Health Services Management and Policy, East Tennessee State University Johnson City, Tennessee, United States of America

Emails

IDO: [ifeoma.ozodiegwu@northwestern.edu](mailto:ifeoma.ozodiegwu@northwestern.edu)

HVD: [doctorh@who.int](mailto:doctorh@who.int)

MQ: [quinnm@etsu.edu](mailto:quinnm@etsu.edu)

LDM: [lmercer@path.org](mailto:lmercer@path.org)

OEO: [Omoike@etsu.edu](mailto:Omoike@etsu.edu)

HMM: [mamudu@etsu.edu](mailto:mamudu@etsu.edu)

Corresponding author: Ifeoma D. Ozodiegwu

Email: [Ifeoma.ozodiegwu@northwestern.edu](mailto:Ifeoma.ozodiegwu@northwestern.edu)

Table of Contents

[Missing Data Imputation 3](#_Toc30255542)

[Logistic Regression Model for Effect Modification Analysis 5](#_Toc30255543)

[Analysis of Credibility 6](#_Toc30255544)

[Intrinsic credibility 6](#_Toc30255545)

[Derivation of basic Critical Prior Interval/SL results 6](#_Toc30255546)

[References 9](#_Toc30255547)

[Acknowledgement 10](#_Toc30255548)

# **Missing Data Imputation**

The fully conditional specification (FCS) method is a sequential iterative algorithm for imputing missing data in a monotone pattern, that is missing data that predominate at the end of a data frame when reading from left to right with no gaps interspaced between full and missing data [1, 2]. Exploration of the study data indicated that our missing data pattern with SAS PROC MI procedure resembled a monotone pattern. An example from the Burkina Faso dataset is provided below:


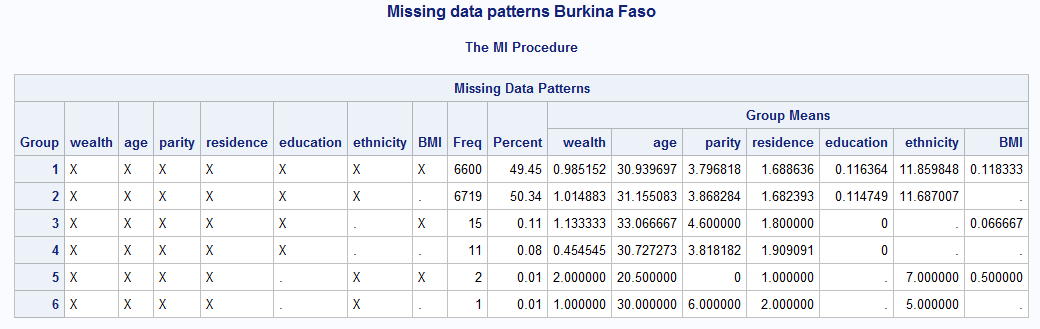


In each iteration of the imputation, the FCS algorithm uses the current values of the observed and imputed values of the imputation model to model the conditional distribution of the missing values for the variables under consideration [2], which in our study was level of educational attainment, ethnicity and BMI. All variables to be imputed were categorical variables and were in the same format as they would be used in the final analysis.

The discriminant function method, one of the imputation methods for classification variables, under the FCS algorithm was used to impute the data in this study using a similar method as described in the SAS software documentation [3].

In summary, to impute data for a categorical variable,$Y_{i}$ , with $k$ responses, we first draw a pooled covariance matrix, $S_{*}=\frac{1}{n-g}\sum_{t=1}^{g} \left( n_{t}-1 \right)S_{t}$ from its posterior distribution. Where $S_{t}$ is the within-group covariance matrix, $n_{t}$ is the group-specific sample size, and $n$ is the total sample size.

Next, for each missing data group, group means, $m_{*t},$ are sampled from the observed group means, $\bar{X}_{t}$, and also from the earlier drawn pooled covariance matrix.

For each group, prior probabilities, $q_{*t}$, of group membership are sampled using a noninformative Dirichlet prior (Jeffreys prior) with $\alpha$= 0.5. The posterior probabilities of an observation are then computed using the group means, $m_{*t},$ the pooled covariance matrix, $S_{*}$ and prior probabilities, $q_{*t}$, as

$$p_{t}\left( x \right)= \frac{exp\left( -0.5D_{t}^{2}\left( x \right) \right)}{\sum_{u=1}^{g} exp\left( -0.5D_{u}^{2}\left( x \right) \right)}$$

Where

$D_{t}^{2}\left( x \right)=\left( x- m_{*t} \right) S_{*}^{-1}2 log\left( q_{*t} \right)$ is the generalized squared distance from $x$to group $t$

Finally, a random uniform variate, $z$, between 0 and 1 is drawn for each missing observation within each group. The discriminant function imputes a range of values for $Y_{i}$ between 0 and 1 depending on the magnitude of $z$compared to the computed posterior probability.

# **Logistic Regression Model for Effect Modification Analysis**

Country level logistic regression models were constructed for the effect modification analysis in the form

$$In \left( \frac{\pi_{i}}{1- \pi_{i}} \right)= \alpha+\sum{BX}_{i}$$

Where $\pi_{i}$is the probability of the being overweight for the $ith$individual in a representative sample of adult women in a particular country, $\alpha$ is the intercept and ${BX}_{i}$ is a vector of model coefficients and covariates, which varied depending on individual characteristics.

For instance, the log odds of overweight in Nigeria woman with the following characteristics – middle income, secondary education, 33 years, Igbo ethnicity, urban residence and 2 children – will be modeled as follows

$In \left( \frac{\pi_{i}}{\pi_{i}} \right)= \alpha+\beta_{1}*1+ \beta_{2}*1+ \beta_{3}*33+ \beta_{4}*1+ \beta_{5}*1+ \beta_{6}*2+ \beta_{7}*1*1$

Where$\alpha=intercept, \beta_{1}=coeffient for middle income household \left( HH \right) wealth, \beta_{2}=coefficient for secondary or higher education \left( SHE \right), \beta_{3}=coefficient for age, {\beta_{4}=coefficient for igbo ethnicity, \beta}_{5}= coefficient for urban residence , \beta_{6}=coefficent for parity , \beta_{7}= coefficient for the interaction between middle income HH and SHE$

# **Analysis of Credibility**

## Intrinsic credibility

In our study, we use the concept of intrinsic credibility, as elaborated by Matthews [4], to assess the credibility of unprecedented statistical significant and non-significant findings. Statistically significant findings are approached with “*fair-minded skepticism*” and non-significant findings with “*fair-minded advocacy*”. The skeptic believes that the most probable value is no effect while the advocate believes that there is an effect.

Operationally, this implies computing critical prior intervals (CPI), which when combined with the statistically significant and non-significant findings includes or excludes effect. Since the evidence used to assess the credibility of unprecedented findings is the central estimate and the confidence intervals from the study alone, findings that lack intrinsic credibility are those with broad CPIs that include the central estimate and vice versa for intrinsically credible findings.

To compute skepticism and advocacy CPIs, we use the formula below provided by Mathews in [5], applying it to the situation where the hypothesis under investigation , which is an OR < 1. The skepticism CPI is in the form (SL, 1/SL), and the advocacy CPI is in the form (AL, 1). Derivations of the CPI are also presented below.

Formulae

$$Skepticism Limit (SL)=\exp\left[ \frac{{In}^{2}\left( \frac{U}{L} \right)}{\sqrt[4]{In \left( U \right)In\left( L \right)}} \right]$$

$$Advocacy Limit= \frac{1}{\exp\left[ \frac{In\left( UL \right) {In}^{2}\left( \frac{U}{L} \right)}{2 In \left( U \right) In \left( L \right)} \right]}$$

## Derivation of basic Critical Prior Interval/SL results

The outcome of the study is modelled via a Normal distribution *N* (M_D,_ V_D_) with the results stated in the conventional format of a 95% CI${(L}_{D}, U_{D})$ so that

$$L_{D}=M_{D}- 1.96\sqrt{V_{D}}\mathrm{and}U_{D}=M_{D}+ 1.96\sqrt{V_{D}}$$

Using the same model for the existing – “prior” - evidence, Bayes’s Theorem shows that combining the new data with prior evidence modelled with *N*(M_o,_ V_o_) leads to an updated or “posterior” distribution *N(*$M_{P}, V_{P})$ whose mean and variance satisfy [6].

$$1/{V_{P}}=1/{V_{D}}+1/{V_{o}}$$

$${M_{P}}/{V_{P}}={M_{D}}/{V_{D}}+{M_{o}}/{V_{o}}$$

where subscripts *p, D* and *o* represent posterior, data (aka likelihood) and prior.

We now apply the Principle of Fair-Minded Challenge. If we have a result claimed to be statistically significant (that is, the 95% CI (L_D,_ U_D_) *excludes* no effect), we challenge it by asking “What level of *prior* evidence is needed such that, when combined with the new evidence from the study, the resulting posterior interval still just excludes no effect, thus making the result credible in the light of that prior evidence?”. We’re then able to examine the resulting prior interval to see if it can be supported by prior (i.e. existing) insight and knowledge of the effect being claimed.

Thus we’re after the prior capable of leading to a posterior distribution such that$L_{P}=M_{P}- 1.96\sqrt{V_{P}}$ = 0; that is, the posterior distribution just touches no effect when the prior evidence is combined with the new result.

From the above we have *M_p_ = V_p_[(M_D­_/ V_D_) + (M_o_/V_o_)] = V_p_[(M_D­_/ V_D_)]* as M_o_ = 0 (because the prior is *skeptical*, and thus has its peak at no effect) and *1/V_p_ = 1/V_o_ + 1/V_D._* It’s *V_o_* that we’re after, because it gives us the width of the skeptical prior interval, centered on M_o_ (= 0) and thus the range of prior values – the *Skeptical Limit (SL) -* capable of making the posterior resulting from the new finding just credible. So

$$M_{P}=\left\{ \frac{1}{\mathrm{Vo}}+\frac{1}{\mathrm{Vd}} \right\}^{-1}\left( \frac{\mathrm{Md}}{\mathrm{Vd}} \right)$$

which when plugged into our constraint on what the posterior credible interval must be gives

$$L_{P}=\left\{ \frac{1}{\mathrm{Vo}}+\frac{1}{\mathrm{Vd}} \right\}^{-1}\left( \frac{\mathrm{Md}}{\mathrm{Vd}} \right)- 1.96\sqrt{\left\{ \frac{1}{\mathrm{Vo}}+\frac{1}{\mathrm{Vd}} \right\}^{-1}}=0$$

We solve this equation for V_o_ to get the link with SL,

$$V_{o}=\frac{\mathrm{Vd}^{2}}{\left( \frac{\mathrm{Md}}{1.96} \right)^{2}-Vd}$$

For the prior we have$U_{o}=M_{o}+ 1.96\sqrt{V_{o}}$ . As by definition this “challenge” prior for a significant result is skeptical it is centered on $M_{o}=0$so the SL leading to a Critical Prior Interval (SPL) of (-SL, +SL) where

$$SL= 1.96\sqrt{V_{o}}$$

and thus, substituting for V_o_:

$$SL=\frac{1.96Vd}{\sqrt{\left( \frac{\mathrm{Md}}{1.96} \right)^{2}-Vd}}$$

Substituting for the data-derived values M_D_ and V_D_ using

$$L_{D}=M_{D}- 1.96\sqrt{V_{D}}\mathrm{and}U_{D}=M_{D}+ 1.96\sqrt{V_{D}}$$

then completes the derivation of the SL to be used for statistically significant results.

As log-normal models are often used in (e.g.) RCTs, we need the log-transformed result for the case of ratios follows analogously, where L­_D_ $\to$ ln (L­_D_) etc. leading to

$$SL=exp\left[ \pm\frac{\ln^{2}\left( U_{D}/L_{D} \right)}{4\sqrt{ln(U_{D})ln(L_{D})}} \right]$$

The results for the case of challenging non-significant results follows from the Principle of Fair-Minded Challenge for that specific case, plus similar algebra to the above.

**References**

1. Berglund PA. Multiple Imputation Using the Fully Conditional Specification Method: A Comparison of SAS®, Stata, IVEware, and R. SAS Institute; 2015. p. 1–17. https://support.sas.com/resources/papers/proceedings15/2081-2015.pdf. Accessed 17 Jun 2018.

2. Berglund PA. An Introduction to Multiple Imputation of Complex Sample Data using SAS® v9. 2010.

3. SAS Institute. PROC MI: Discriminant Function Method for Monotone Missing Data :: SAS/STAT(R) 9.2 User’s Guide, Second Edition. https://support.sas.com/documentation/cdl/en/statug/63033/HTML/default/viewer.htm#statug_mi_sect022.htm. Accessed 15 Jan 2020.

4. Matthews RAJ. Beyond ‘significance’: principles and practice of the Analysis of Credibility. R Soc Open Sci. 2018. doi:10.1098/RSOS.171047.

5. Matthews RAJ. Moving Towards the Post p < 0.05 Era via the Analysis of Credibility. Am Stat. 2019;73:202–12. doi:10.1080/00031305.2018.1543136.

6. Lee P. Chapter 1. Bayesian Statistics. 2012.

# **Acknowledgement**

We thank Dr. Matthews for providing support in elucidating the derivations of the Critical Prior Intervals.
